# Alcohol Use Patterns Among Urban and Rural Residents: Demographic and Social Influences

**DOI:** 10.35946/arcr.v38.1.09

**Published:** 2016

**Authors:** Mark A. Dixon, Karen G. Chartier

**Affiliations:** Mark A. Dixon, L.C.S.W., is a doctoral student in the School of Social Work, and Karen G. Chartier, Ph.D., M.S.W., is an assistant professor in the School of Social Work and the Department of Psychiatry, both at Virginia Commonwealth University, Richmond, Virginia

**Keywords:** Alcohol use, abuse, and dependence, alcohol use patterns, alcohol use disorder, geographic location, urban society, rural society, risk and protective factors, demographic risk and protective factors, cultural risk and protective factors, environmental risk and protective factors, social influences

## Abstract

Rates of alcohol use and alcohol use disorder (AUD) vary with geographic location. Research on risks for AUD associated with living in a rural versus urban setting is complicated by the varied systems used to classify geographic location. Studies comparing the prevalence of heavier or binge drinking and AUD based on a dichotomous urban/rural classification have mixed findings when compared with those using more detailed urban-to-rural categories. In addition, urban/rural residence interacts with other demographic factors such as age, U.S. region, and race/ethnicity to affect alcohol use. Social and cultural factors help explain the relationship between geographic location and alcohol use. However, this area of research could be improved by the use of standardized definitions as well as the analysis of a more complete urban-to-rural continuum (e.g., urban, suburban, and rural areas). Having a better understanding of how geographic characteristics influence alcohol use would help inform and improve prevention and treatment efforts.

Geographic location can be an impor-tant factor in determining a person’s level of risk for alcohol-related problems. Certain factors associated with living in an urban or rural area may increase risk, while others may be protective. For example, the availability of alcohol, norms for acceptable drinking behaviors, demographic characteristics, and economic factors all vary with respect to geographic area and may influence drinking behaviors. The National Institute on Alcohol Abuse and Alcoholism’s (NIAAA) Health Disparities Strategic Plan 2009–2013 (NIAAA 2009) recognized that differences exist due to location and called attention to addressing the impacts of alcohol use and its consequences on rural populations. This article represents a partial response to that call and examines rates of alcohol use and alcohol use disorder (AUD) in urban versus rural locations. Consideration is also given to how U.S. region, race/ethnicity, and age intersect with these drinking patterns, as well as other social and cultural factors that characterize place of residence. Both government documents and peer-reviewed journal articles were used to examine this topic. This article considers how more delineated categories on an urban-to-rural continuum could better characterize the relationships between geographic location, alcohol consumption, and AUD and improve prevention and treatment efforts.

## Definitions of Urban versus Rural Population Areas

Defining and characterizing urban and rural population areas can be a complicated task. There are over two dozen definitions of “rural” used by U.S. government agencies (Bucholtz 2008). Three examples of such definitions are presented in table 1. These definitions have been applied in alcohol studies (with some of the related results reviewed in this article) and have implications for defining the percentage of the U.S. population that live in an urban versus a rural area. For example, according to the U.S. Census Bureau (USCB) and using its urban area, urban cluster, and rural area classifications, approximately 80.7 percent of the U.S. population in 2010 lived in an urban community, with the remainder (19.3 percent) living in a rural area (USCB 2013). The Office of Management and Business (OMB) employs a different 3-group urban- to-rural classification (OMB 2010, 2013), which defines Core Based Statistical Areas (CBSA) as metropolitan, micropolitan, or non-core based. The CBSA classification has been used to define a rural area in two ways: (1) living outside of both a metropolitan and a micropolitan county, or (2) only living outside of a metropolitan county. Based on these two definitions, in 2010 approximately 6.3 percent or 16.3 percent of Americans, respectively, lived in a rural area (Mackun and Wilson 2011). The United States Department of Agriculture (USDA), through the Economic Research Service (ERS), has also developed multiple methods of categorizing non-metropolitan counties, one of which is referred to in table 1 (USDA 2013*b*). According to the USDA denition of metropolitan versus non-metropolitan areas, in 2012, approximately 14.7 percent of the U.S. population lived in a non-metropolitan area (USDA 2013*a*).

These definitions exemplify the potential difficulties involved in defining urban or rural settings, and the possibility of organizing geographic data into categories based on a variety of urban/rural thresholds. These varied definitions complicate the study of how urban and rural areas are associated with patterns of alcohol use in the United States. For example, population estimates of alcohol use and AUD from the Substance Abuse and Mental Health Services Administration annual household surveys (from 1971 to 2001 called the National Household Survey on Drug Abuse [NHSDA], and from 2002 to the present called the National Survey on Drug Use and Health [NSDUH]) cannot be readily compared across urban and rural categories. The NHSDA defined urban and rural residence through a dichotomous metropolitan versus non-metropolitan classification using OMB definitions (SAMHSA 2003*a*), whereas the NSDUH uses the expanded 9-category classification based on the Rural/Urban Continuum Codes (RUCC) and updated OMB standards for defining a metropolitan area. Given the periodic updates of these definitions by government agencies, it can even be difficult to compare surveys from year to year (e.g., changes made from the 2002 to the 2003 NSDUH surveys) (SAMHSA 2004).

According to the 2002 NSDUH, prevalence rates of past-year alcohol use were highest for those living in large (72.9 percent) and small metropolitan areas (70.2 percent) compared with non-metropolitan areas (61.6 percent) (SAMHSA 2003*b*). Data from the National Epidemiologic Survey on Alcohol and Related Conditions (NESARC) for 2001–2002 used OMB’s CBSA system to define geographic residence. One report identified past-year alcohol use rates using a dichotomous urban (67.2 percent) versus rural (58.4 percent) delineation (Dawson et al. 2011). Both surveys show higher rates of drinking in metropolitan areas. However, the utility is compromised, because the two surveys do not use consistent definitions and classifications to define place and are not entirely comparable. These surveys do use the same U.S. region classification based on USCB’s state groupings (i.e., Northeast, Midwest, South, and West), enabling region-based estimates to be compared between the surveys.

## Variations in Rates of Alcohol Use and AUD Across the Urban-to-Rural Continuum

Despite these varying definitions, epidemiologic studies have attempted to characterize geographic differences in prevalence rates of alcohol use and AUD (either reporting lifetime or past 12-month AUD rates or rates of alcohol dependence) in the adult U.S. population over the past 20 years. According to data from the 1991–1992 National Longitudinal Epidemiologic Survey (NLAES) (using an older version of OMB’s metropolitan statistical area/non-metropolitan statistical area classification), the residents in urban areas compared with rural areas (odds ratio = 1.22) were more likely to report lifetime alcohol use. Among drinkers, however, urban and rural residents had similar risks for lifetime alcohol dependence (Grant 1997).

Using 2001–2002 NESARC data, Dawson and colleagues (2011) reported, as shown above, that prevalence rates of past-year drinking in the adult population were higher for urban residents compared with rural ones. However, the rates of past-year heavy episodic drinking (i.e., 5 or more drinks on any day for men, and 4 or more drinks on any day for women) were similar for residents living in both locations (23.7 and 23.2 percent for urban and rural residents, respectively). The 12-month AUD rates among urban and rural residents (8.4 percent and 8.8 percent, respectively) were also similar. Another analysis of NESARC data found that the lifetime prevalence of an AUD was somewhat lower for urban residents (29.6 percent) than for rural ones (33.3 percent) (Hasin et al. 2007).

Further, Borders and Booth (2007) used 2001–2002 NESARC data and a 3-tiered (urban, suburban, and rural) classification of residence based on OMB’s CBSA definitions. They found that rates of abstinence were lowest for suburban residents (31.3 percent) compared with urban (35.4 percent) and rural (41.7 percent) residents. However, rural drinkers were significantly more likely than suburban drinkers to report exceeding the recommended daily drinking limits (more than 4 drinks for men and more than 3 drinks for women) (suburban: 34.5 percent; urban: 37.4 percent; and rural: 40.0 percent). Urban drinkers were more likely than suburban drinkers to report drinking more than 14 drinks for men and more than 7 drinks for women in a typical week (i.e., exceeding recommended weekly drinking limits) (suburban: 14.9 percent; urban: 17.1 percent; and rural: 16.7 percent). Rural drinkers (15.1 percent) were also significantly more likely than suburban drinkers (11.6 percent) to report a past-year AUD, with rates for urban drinkers (14.0 percent) falling in between.

The 2011 and 2012 NSDUH (SAMHSA 2013) include more current data, although these findings are not easily comparable with NLAES and NESARC. For adults ages 18 and older in 2011, the prevalence of past 12-month AUD was higher in large metropolitan areas (7.1 percent) and small metropolitan areas (7.0 percent) than in non-metropolitan areas (4.9 percent). In 2012, these rates remained higher for residents in metropolitan areas (large metropolitan: 7.4 percent; small metropolitan: 7.4 percent), but the past 12-month AUD rate for residents in non-metropolitan areas increased from the previous year to 6.1 percent. Recent treatment admissions data, based on the 2009 Treatment Episode Data Set (TEDS), showed other differences by urban and rural locations using, the National Center for Health Statistics (NCHS) standards and based on census data and Metropolitan Statistical Areas (MSAs) (Eberhardt et al. 2001; NCHS 2014). For example, persons admitted to treatment in rural areas (49.5 percent) were more likely to report alcohol as their primary drug of abuse compared with persons admitted in urban areas (36.1 percent) (SAMHSA 2012).

Although these studies are difficult to compare, the ones reviewed here suggest that rates of alcohol use are higher for urban versus rural residents and that rates of AUD tend to be similar across rural and urban environments. However, there is some indication that a more detailed evaluation of the urban-to-rural continuum will yield more nuanced relationships with alcohol use and AUD across geographic areas, particularly when suburban residence is separated from and compared with rural and urban residence.

## Interactions Between Rural/Urban and Other Demographics

To understand an individual’s alcohol- related risk profile, it is important to consider the interaction of a number of demographic characteristics with geographic setting. The sections below examine U.S. region, race/ethnicity, and age as factors that interact with rural/urban setting to influence risk.

### U.S. Regions

The Southern U.S. region consistently has the lowest rates of alcohol use. The 1991–1992 NLAES showed the lowest rates of lifetime drinking among Southern residents, followed by residents of the Midwest, West, and Northeast (Grant 1997). Drinkers in the West and Midwest were more likely than Southern drinkers to report lifetime alcohol dependence, whereas drinkers in the Northeast were less likely to report such dependence compared with those in the South. Similarly, based on survey data from the 1993 Behavioral Risk Factor Surveillance System (BRFSS), residence in the deep South (Alabama, Georgia, Louisiana, and Mississippi) was the single greatest predictor of past-month abstinence compared with other regionally representative states (New York, Illinois, Colorado, and California) (Lindquist et al. 1999). Further analysis of AUD based on the 2001–2002 NESARC showed that the Midwest (35.3 percent) and West (32.6 percent) had higher percentages of residents with a lifetime AUD compared with the Northeast (27.1 percent) and South (27.0 percent) (Hasin et al. 2007). NSDUH data from 2012 also showed that those living in the West had the highest past 12-month AUD rate at 8.0 percent, followed by the Midwest (7.7 percent), Northeast (6.8 percent), and South (6.5 percent). For residents in the South, the 2012 past 12-month AUD rate was significantly higher than in 2011 (5.7 percent), whereas the rates for other U.S. regions showed little change from the previous year (SAMHSA 2013). Researchers suggest that a relationship exists in the South between the high levels of Protestant religiosity, which encourages abstinence, and lower drinking and AUD rates (Booth and Curran 2006; Lindquist et al. 1999; Michalak et al. 2007). Religiosity and other social and cultural factors that are associated with geographic location and alcohol use are reviewed in a later section.

Using 2001–2002 NESARC data, Borders and Booth (2007) examined the intersection between urban, suburban, and rural residence and U.S. regions in predicting alcohol use and AUD. Residents from the rural South were most likely to abstain from drinking; they had the highest past-year abstinence rate at 52.1 percent compared with the next highest rate at 39.0 percent for urban Northeast residents. The lifetime abstinence rate was also highest in the rural South (27.5 percent) but lowest in the rural Northeast (9.2 percent). The urban Midwest (29.4 percent) had the highest percentage of residents exceeding daily drinking limits, and the rural South had the lowest percentage (17.3 percent). Residents in the urban West (18.3 percent) were more likely to exceed weekly drinking limits, whereas residents in the suburban Midwest were least likely to (12.7 percent). Urban Midwest drinkers also reported the highest prevalence of past 12-month AUD (12.4 percent), followed by drinkers in the rural Midwest (11.0 percent) and rural West (10.3 percent). The lowest rate of past 12-month AUD was reported by residents in the rural South (6.7 percent).

These regional urban-to-rural comparisons based on the NESARC set the rural South and the urban Midwest at opposite endpoints of the continuum from less risky to more risky drinking and AUD. The ranking of other locations in between these points is less consistent. Eberhardt and colleagues (2001) examined data from multiple government agencies (CDC, SAMHSA, DHHS) about rural and urban health. They reported within- region comparisons for heavy alcohol use (i.e., 5 or more drinks in one day) between metropolitan and non- metropolitan residents using MSAs. For example, in both the Northeast and West, adults ages 18 to 49 who lived in small metropolitan and non- metropolitan areas had higher rates of past-year heavy drinking than those who lived in large metropolitan areas within those same regions. It was also found that men in metropolitan areas were more likely to engage in heavy drinking (56 percent) compared to non-metropolitan areas (48 to 52 percent). However, it is unclear to what degree including a well-defined suburban classification would have altered the results.

### Race and Ethnicity

The intersection of race and ethnicity with urban and rural location is another important comparison for understanding the alcohol use patterns of U.S. subpopulations. Data from several different reports generated using 2010 census data reveal concentrations of racial/ethnic groups across certain geographic areas (Ennis et al. 2011; Hixson et al. 2011, 2012; Hoeffel et al. 2012; Norris et al. 2012; Rastogi et al. 2011). The U.S. population of rural residents has shifted some; for example, the percentage of Hispanics living in rural areas has increased (in 1980, 3 percent; and in 2006, 6 percent) (Economic Research Service, n.d.). Rural residents in 2012 were 78 percent White, 9 percent Hispanic, and 8 percent Black, while urban residents were 44 percent White, 27 percent Black, and 17 percent Hispanic (Housing Assistance Council 2012). American Indian reservations are often in rural areas; however, only 22 percent of American Indians/Alaska Natives live on a reservation, on trust land, or in other designated areas (Norris et al. 2012).

Some studies examining the rates of alcohol use and AUD among race/ethnic groups by urban and rural location have mixed results. Booth and Curran (2006) studied Blacks and Whites in six Southern states and showed that rural residence (i.e., living outside of an MSA) was protective for alcohol use in both Blacks and Whites. Urban Blacks had higher abstinence rates (63.0 percent) than urban Whites (49.9 percent) over the past 28 days, while rural residents of both groups had similar abstinence rates (66.8 percent and 65.5 percent, respectively). Blacks in urban areas also had lower rates of current problem drinking compared with Whites in urban areas (6.1 percent versus 10.0 percent), but similar rates to Whites in rural areas (6.0 percent and 6.9 percent, respectively). Diala and colleagues (2004) examined lifetime AUD rates across urban-to-rural locations for Blacks and Whites using the 1990–1992 National Comorbidity Survey. Blacks were less likely than Whites to report a lifetime AUD in rural areas (i.e., counties with less than 2,500 population) and urban areas (i.e., counties with a city of 50,000 or more population), but both groups had a similar likelihood in large metropolitan areas (i.e., counties with 100,000 or more population and a central city). Differences in the findings between these two studies may be attributed to the different definitions of urban/rural residence used by each study or the samples: Southern residents versus U.S. adults.

Using 2003 NSDUH data, Van Gundy (2006) compared past 12-month AUD rates for several races/ethnicities by urban versus rural location in two age groups. For young adults age 18 to 25, Whites were significantly more likely to report an AUD when living in an urban area (i.e., metropolitan area; 20.0 percent) versus a rural one (i.e., non-adjacent metropolitan area; 17.9 percent). The rates among Blacks in that age group were similar in urban (9.9 percent) and rural environments (10.5 percent). AUD rates declined with older age for all racial and ethnic groups. Among Blacks age 26 and older, those in urban areas had significantly higher rates (6.8 percent) of AUD compared with those in rural areas (3.0 percent). The difference in AUD rates among Whites was less dramatic ranging from 6.2 percent (urban) to 5.5 percent (rural). The AUD rate for Whites was similar to that of Blacks in urban areas in this 26-and-older age group; yet in rural areas, AUD rates were lowest for Blacks compared with other racial/ethnic groups. AUD rates were not significantly different among Hispanics or Asians/Pacific Islanders by urban or rural setting in either the 18-to-25 age group (Hispanics: 15.3 percent urban, 15.0 percent rural; and Asians/Pacific Islanders: 14.4 percent urban, 20.2 percent rural) or the 26-and-older age group (Hispanic: 6.6 percent urban, 8.3 percent rural; and Asians/Pacific Islanders: 3.6 percent urban, 5.8 percent rural). Bigger sample sizes could be needed to identify significant differences in some of these race/ethnicity-by-age subgroups.

Van Gundy (2006) also reported no significant differences in the 12-month AUD rates between American Indians living in urban and rural areas, either for individuals ages 18 to 25 (urban 24.9 percent; rural 20.2 percent) or ages 26 and older (urban 16.6 percent; rural 13.9 percent). An earlier study suggested that there is little difference in the quantity of alcohol consumed by urban and rural American Indians, but that urban American Indians tend to drink more frequently (Weisner et al. 1984). Other studies have examined alcohol use for American Indians living in different U.S. regions, including the Southwest and Plains regions that comprise parts of the West, Midwest, and South. O’Connell and colleagues (2005) examined drinking patterns across four groups: (1) reservation-based Southwestern Indians (SW-AI); (2) reservation- based Northern Plains Indians (NP-AI); (3) American Indians who were geographically dispersed (NLAES-AI); and (4) the U.S. general population excluding American Indians (NLAES- GP). Sixty percent of the NLAES-AI group lived in urban areas, while the reservation-based American Indian groups were primarily rural residents (O’Connell et al. 2005). Comparisons of American Indians living on and off reservation areas overlap some with rural versus urban comparisons; however, rural reservations have unique characteristics not shared with rural areas more generally. Reservation- based American Indians (SW-AI and NP-AI) showed a general pattern not only of high-quantity drinking (e.g., higher rates of drinking 5 or more drinks in 1 day and being intoxicated in the past year), but also of low- frequency drinking (e.g., lower rates of drinking monthly and drinking more than 8 days in a month). NP-AI males and females, in particular, were most likely to report high-quantity drinking. Several studies report that American Indians are less likely than the general U.S. population to be current drinkers; however, there is variability in the drinking rates and quantity of consumption by region and tribal affiliation (Beauvais 1998; May 1996; Szlemko et al. 2006; Young and Joe 2009).

### Underage Drinking in Urban and Rural Areas

Using NSDUH data, rates of underage drinking can be compared across urban-to-rural locations. Pemberton and colleagues (2008) reported on past-month alcohol use and binge drinking based on the 2002–2006 NSDUH for 12- to 20-year-olds. County types were categorized by a 4-level urban-to-rural continuum, including metropolitan areas both large (with a population of 1 million or more) and small (less than 1 million population), as well as urbanized (20,000 or more population) and rural (less than 20,000) non-metropolitan areas. Past-month alcohol use was similar across location categories— i.e., large metropolitan (27.5 percent), small metropolitan (30.1 percent), urbanized non-metropolitan (31.3 percent), and rural non-metropolitan (28.1 percent). Prevalence rates for binge drinking were also similar by location (large metropolitan 17.7 percent; small metropolitan 20.8 percent; urbanized non-metropolitan 22.2 percent; and rural non-metropolitan 19.8 percent). Conversely, Lambert and colleagues (2008) used 2002–2004 NSDUH data for individuals ages 12 to 17 and reported significantly higher rates of past-month alcohol use and binge drinking when comparing four rural categories to one combined metropolitan category. These rates were highest in the most rural category (i.e., medium to small rural areas with a population less than 20,000 and not adjacent to a metropolitan area). Findings were less consistent for young adults ages 18 to 25 when comparing rural and urban areas.

Table 2 presents urban/rural prevalence rates based on 2002–2006 NSDUH data for Whites, Blacks, and Hispanics between ages 12 and 20 (Pemberton et al. 2008). In metropolitan areas, underage Whites were more likely to engage in binge drinking than Hispanics, while in urbanized non- metropolitan areas the rates between Whites and Hispanics were similar, and in rural non-metropolitan areas Hispanics had higher rates than Whites. Comparable differences were observed for rates of past-year AUD between Whites and Hispanics across urban/rural areas. Underage Blacks had higher rates of binge alcohol use and past-year AUD in urbanized non-metropolitan areas than in other areas; however, prevalence rates of binge drinking and AUD were lower for Blacks than Whites and Hispanics, regardless of urban/rural category.

Past-year AUD rates, reported by Van Gundy (2006) and based on the 2003 NSDUH, included additional race/ethnic groups. Comparisons were made based on an urban and rural dichotomy and in a smaller age group of youth ages 12 to 17. These data seem to similarly distinguish rural Hispanic youth as a potential risk group. Hispanics who live in rural areas (8.9 percent) were significantly more likely to report an AUD than those who live in urban areas (4.9 percent). Asian/Pacific Islanders reported higher rates of AUD in rural (11.4 percent) compared with urban (4.1 percent) areas, but this difference did not reach statistical significance. All other ethnic groups (i.e., Whites, Blacks, and American Indians/Alaska Natives) reported similar past-year rates of AUD in urban and rural areas.

## Beyond Rural vs. Urban: Social and Cultural Characteristics of Geographic Locations

Understanding the relationship between alcohol use and geographic location requires more than assessing population density and proximity to a metropolitan area. A number of social and cultural factors are related to alcohol use patterns and also characterize urban and rural settings. These include religious cultural practices, community and family relationships, economic conditions, the availability of alcohol, and the enforcement of alcohol laws, among others. One mechanism that links these characteristics to drinking is the potential to control (increase or decrease) access to alcohol for residents in an area, but they may alternatively represent potential buffers or stressors that influence alcohol use.

Social relationships in a community may influence drinking behaviors. As previously mentioned, lower alcohol use rates in the Southern states have been attributed to higher participation in religions that encourage abstinence. A 2000 National Alcohol Survey study found that higher levels of religiosity and the religious proscription of drinking are significantly associated with drinking behaviors, particularly higher abstinence levels (Michalak et al. 2007). Community social capital, defined as neighborhood attachment, supportiveness, or participation, is also protective for problem drinking (Bryden et al. 2013). The family environment in particular, including parental monitoring, parental approval, and communication style, has a strong influence on drinking patterns among youth (Nash et al. 2005). Van Gundy (2006), for example, reported a 4-percent increase in alcohol abuse among rural youth when either the mother or father were absent from the home.

The economic conditions in a geographic area may be associated with local rates of alcohol use. Karriker-Jaffe (2011) reported varied relationships between alcohol outcomes and area-level socioeconomic status. Neighborhood disadvantage was associated with more heavy alcohol use in adults, while neighborhood advantage was associated with more alcohol use among underage drinkers. The qualities of the built environment, where someone lives, are also associated with alcohol use. Bernstein and colleagues (2007) reported that residents living in urban areas characterized by substandard buildings (stairway, window, or heating problems) were more likely to report heavy drinking. Community disorder more generally, defined by population density, crime, etc., was positively associated with alcohol use in adolescents and adults (Bryden et al. 2013).

Both the perceived and actual availability of alcohol from formal and informal sources can influence the prevalence of drinking and related problems (Treno et al. 2008). In adolescents, greater exposure to alcohol advertising was associated with increased drinking and a greater likelihood of alcohol use (Bryden et al. 2012). In assessing the relationship between alcohol outlet density (AOD) and specific area-level demographic characteristics, Berke and colleagues (2010) examined urban, suburban, large town, and rural geographic locations. In urban areas, AOD was associated with poverty, education, and Black and Hispanic race/ethnicity, but there were no associations for these characteristics with AOD in suburban areas, large towns, and rural areas. AOD predicted higher rates of binge drinking in urban areas at densities greater than 80 alcohol outlets per square mile (Ahern et al. 2013). The retail mix in a geographic area may also matter (i.e., higher binge drinking rates were reported in areas with liquor stores only versus areas with food stores only) (Shimotsu et al. 2013).

Other means of controlling the availability of alcohol in a geographic area include alcohol taxation and the enforcement of alcohol laws. There is evidence to support the use of price and tax policies; higher alcohol prices and taxes are associated with reductions in problems associated with binge and heavy drinking, including alcohol-related crash facilities (Elder et al. 2010). Jackson and colleagues (2014) reported that both the perceived enforcement of liquor laws and the level of funding for enforcement are associated with lower levels of alcohol use. Paschall and colleagues (2012) similarly showed that funding for underage drinking enforcement across various size cities in California was associated with a lower frequency of alcohol use in adolescents, but that AOD and the level of adult drinking in the area had positive correlations with adolescent drinking. Finally, Ying and colleagues (2013) recommended, to be most effective, that alcohol laws and policies (e.g., zero tolerance, open container, minimum legal drinking age, and blood alcohol content) should be adapted to the characteristics of the area where they are implemented.

### Implications for Prevention and Treatment

The urban/rural patterns of alcohol use and area-level characteristics described above may have implications for developing intervention strategies. First, the reviewed research identifies potential at-risk subpopulations to target for intervention. Urban residents showed lower rates of abstinence; but more specifically, Midwest residents in urban areas had higher rates of heavier drinking and AUD. By both race/ethnicity and age, there was some evidence that White young adults and older Black adults had higher AUD rates in urban areas. Conversely, rural residence was associated with higher AUD rates for underage Hispanic drinkers, and underage drinking appeared to be higher in the most rural U.S. areas. American Indians had high AUD rates in both urban and rural settings, but reservation-based American Indians in the Northern Plains were at greater risk.

Second, the reviewed research may suggest potential strategies for reducing risky alcohol use in a geographic area, including at individual, community, and policy levels. For example, knowledge of the level of religiosity, the community and family relationships, and the social drinking norms of a population could be used to further target at-risk groups or to conceptualize intervention and prevention strategies. A computerized training program for 12-year-olds living in an urban setting showed positive effects (e.g., lower alcohol use and binge drinking and fewer drinking friends) that held over the course of 7 years compared to the control group (Schinke et al. 2010). Though not specifically addressed, this may have implications for rural underage drinking reduction; computerized intervention methods may be a cost-effective option for rural and sparsely populated areas. Geographic areas characterized by greater socioeconomic disadvantage and disorder could be targeted for community-level interventions to address these conditions and to reduce problem alcohol use through the building of social capital. Policy-level interventions to reduce AOD or to change the mix of retail options in a community may be of particular importance in urban areas, while alcohol taxation and law enforcement are more generally effective at reducing heavy drinking and drinking-related problems across geographic locations.

It also is important to consider whether the availability of treatment services matches the need in urban and rural areas. Lenardson and Gale (2007) used data from the 2004 National Survey of Substance Abuse Treatment Services to comparatively describe treatment facilities in urban and rural locations. Fewer facilities and treatment beds are located in rural areas. Approximately 9 percent of all surveyed treatment facilities were located in a non-metropolitan area that is not adjacent, 12 percent in an adjacent non-metropolitan area, and 79 percent in a metropolitan area. Differences in the types of services offered by treatment facilities in urban and rural locations may also influence access to treatment services. Lenardson and Gale (2007) also reported that non-metropolitan treatment facilities were less likely than metropolitan ones to offer detoxification (15.4 percent versus 22.4 percent), transitional housing (7.6 percent versus 10.9 percent), and day treatment/partial hospitalization programs (9.4 percent versus 15.2 percent). Non-metropolitan counties also had a lower percentage of facilities offering substance abuse specialty services (51.9 percent) compared with metropolitan facilities (64.3 percent). It is unclear to what extent that the treatment needs in rural and urban areas are or are not being met according to this reported availability of services. However, given that the reviewed studies showed similar rural and urban AUD rates or higher rates among some segments of the rural population, it seems inconsistent that the need for treatment would be less in rural areas than urban ones. This apparent discrepancy between treatment availability and treatment need in rural areas could require a policy- level intervention.

### Recommendations

Conducting alcohol studies on urban and rural populations is complicated by the various methods of defining these terms. The definitions have changed over time and are different across surveys, complicating direct comparisons between studies. Consistent and clearly stated definitions of what is meant by urban, suburban, or rural are important for understanding the relationship of these geographic locations to drinking patterns, as well as their implications for prevention and treatment needs. A dichotomous urban/rural classification may inappropriately aggregate data such that it masks the risky drinking behaviors of populations living in urban or rural areas compared with suburban locations. Future studies need to go beyond a rural/urban dichotomy to more fully examine the urban-to-rural continuum. For example, Kuo and Porter (1998) completed a demographic study and examined seven subgroups of Asian/Pacific Islanders in urban, suburban, and rural areas and across regions. Borders and Booth (2007) also offer an example of how to examine alcohol use patterns by intersecting regional and urban, suburban, and rural locations. Further study of differences in drinking and risks for AUD across the urban- suburban-rural continuum could present a more contextualized understanding of the relationship between alcohol use and geographic context.

## Figures and Tables

**Table 1:** Three Classifications of Urban-to-Rural Geographic Locations

Government Agency	Primary Geographic Area	Basis of Classification	Urban-to-Rural Categories
U.S. Census Bureau (USCB)	Census tract	Population density	Three-tier classification system: (1) Urban areas are census tracts with populations of 50,000 people or more; (2) urban clusters are census tracts with populations from 2,500 to 49,999; and (3) rural areas are all other census tracts outside urban areas and urban clusters.^1^
Office of Management and Budget (OMB)	County	Population clusters; and urbanized cores	Counties are designated as a Core Based Statistical Area (CBSA) or a non-CBSA area. CBSA areas are subdivided into Metropolitan Statistical Areas (MSA), or counties with an urbanized core of 50,000 residents or more; and Micropolitan Statistical Areas, or counties with a population cluster of between 10,000 and 49,999 residents. Frequently, MSA is used when discussing this classification system rather than CBSA.^2^
U.S. Department of Agriculture (USDA), and Economic Research Service (ERS)	County	Rural/Urban Continuum Codes (RUCC)	OMB’s Metropolitan/non-Metropolitan Statistical Area categories are further divided. Metropolitan Statistical Areas are divided into three subcategories based on USCB population estimates; and non-metropolitan (i.e., Micropolitan Statistical Area and non-CBSA area) are divided into six subcategories, based on proximity to a Metropolitan Statistical Area. Metropolitan subcategories include (1) metro counties of 1 million population or more; (2) metro counties of 250,000 to 1 million; and (3) metro counties of less than 250,000. Non-metropolitan subcategories include: (1) non-metro county with urban population of 20,000 or more adjacent to a metro area; (2) non-metro county with urban population of 20,000 or more not adjacent to a metro area; (3) non-metro county with urban population between 2,500 and 19,999 adjacent to a metro area; (4) non-metro county with urban population between 2,500 and 19,999 not adjacent to a metro area; (5) rural county with urban population less than 2,500 adjacent to a metro area; and (6) rural county with urban population less than 2,500 not adjacent to a metro area.^3^

NOTE: Urban-to-rural classifications were based on information from the following sources:

1 USCB 2012;

2 OMB 2010, 2013; and

3 USDA 2013*a*,*b*.

**Table 2:** Prevalence of Underage Binge Drinking and Alcohol Use Disorder (AUD) by Urban to Rural Area and Race/Ethnicity (Percentage)

	Metropolitan Area*	Urbanized Non-metropolitan Area	Rural Non-metropolitan Area
**Binge Alcohol Use**			
Whites	22.9	23.6	20.7
Blacks	9.0	14.2	10.4
Hispanics	17.0	21.1	24.7

**AUD**			
Whites	10.9	12.1	10.0
Blacks	4.4	7.8	4.9
Hispanics	8.4	11.3	12.5

NOTE:

* Metropolitan included both large and small metropolitan areas. Percentages were from the 2002–2006 NSDUH for youth ages 12 to 20 (Pemberton et al. 2008). Binge alcohol use was in the past 30 days and alcohol use disorder in the past year.
